# Development of a Fully Flexible Sheet-Type Tactile Display Based on Electrovibration Stimulus

**DOI:** 10.3390/mi9050230

**Published:** 2018-05-11

**Authors:** Hiroki Ishizuka, Ryuhei Hatada, Carlos Cortes, Norihisa Miki

**Affiliations:** 1 Department of Intelligent Mechanical Systems Engineering, Kagawa University, 2217-20 Hayashi-cho, Takamatsu, Kagawa 761-0396, Japan; 2 Department of Mechanical Engineering, Keio University, 3-14-1 Hiyoshi, Kohoku-ku, Yokohama, Kanagawa 223-8522, Japan; hahaharyuhe1@gmail.com (R.H.); cortestc@keio.jp (C.C.); miki@mech.keio.ac.jp (N.M.)

**Keywords:** tactile displays, electrovibration stimulus, flexible device, thermal stimulus, multiple stimulus

## Abstract

Tactile displays have been extensively studied for several decades. However, owing to their bulkiness and stiffness, it has been difficult to integrate these displays with information devices to enable tactile communication between the devices and their users. This paper proposes a novel sheet-type electrovibration tactile display that consists of poly(3,4-ethylenedioxythiophene) polystyrene sulfonate conductive layers and an insulation layer of polydimethylsiloxane. The tactile display is sufficiently thin and flexible for attaching onto various surfaces. In this study, the tactile display was micro-fabricated and characterized through experiments. The experimental results indicated that the tactile display exhibited good durability under bending and that it could present various tactile sensations depending on the type of voltage waveform. In addition, the effect of using a combination of electrovibration and thermal stimuli was also demonstrated. The sheet-type display was attached onto a Peltier element; the thinness of the structure enabled the display to conform to the element and ensure good heat transfer. In the experiment, subjects were asked to scan the display with their fingertips. The results showed that multiple tactile stimuli were also successfully perceived by the subjects.

## 1. Introduction

Tactile displays, which can present pseudo-tactile sensation, have been studied for several decades. Recently, tactile displays have attracted industrial interest, since tactile feedback can improve not only the controllability of information devices, but also the quality of the contents presented using the devices. These tactile displays are mainly categorized into mechanical and electrical types. The mechanical tactile displays directly deform the skin with a single actuator or an array of actuators. For example, Lévesque et al. developed a mechanical tactile display with an array of piezo actuators. They experimentally confirmed that the proposed tactile display was able to present stimulus patterns such as a circle or a triangle [1]. Choi et al. developed a tactile display with an electroactive polymer [2], while Gallo et al. fabricated a flexible multimodal tactile display using microfabrication process [3]. The tactile display consisted of an array of magnetic actuators for bumpy stimulus, and heating circuits for thermal stimulus. Hoshi et al. developed a tactile display that converted ultrasound radiation into skin deformation [4]. This principle was combined with pneumatic stimulus and femtosecond laser stimulus [5,6]. The squeeze film effect generated by the ultrasonic vibration can reduce the frictional force on physical surfaces. Biet et al. developed a tactile display based on the squeeze film effect [7]. In sensory evaluation, the subjects perceived slippery sensation via the tactile display. Microelectromechanical systems (MEMS) technology is beneficial in miniaturizing the actuators, and thus enhancing the resolution of tactile displays [8]. An array of large-displacement microactuators composed of piezoelectric actuators and hydraulic displacement amplification mechanisms enable a variety of tactile sensations [9,10,11]. The electrical tactile displays directly stimulate the nerves inside the human skin using electric current. Kajimoto developed a cylindrical tactile display containing 1536 electrodes, aimed at stimulating the entire hand [12]. Kitamura et al. fabricated an array of needles for electrical tactile stimulus with wire tip etching [13]. The needle-type electrical tactile display was able to present tactile stimulus with low voltage, since the tactile display penetrated the epidermis and applied the voltage directly to the dermis [14]. Tezuka et al. experimentally proved that the needle-type electrotactile display can control the roughness of virtually presented surfaces, and that it can also be used as a tactile communication tool by attaching it to the forearms [15]. It may be noted that almost all these tactile displays are designed to be used alone. Tactile displays have the potential to add tactile feedback functionality to existing information devices, however, this is hindered by their stiffness [1,2,3,9,10,11,12] and bulkiness [4,5,6,7]. The display consequently increases the volume and weight of the information devices. The high stiffness restricts the shape of the surface where the tactile displays are mounted. There are several studies that demonstrate flexible tactile displays [16,17]. However, these tactile displays are not perfectly thin and flexible, since these tactile displays are not sufficiently thin to be bent easily. Further, the mechanical parts of these tactile displays may break under an external impact. Thus, to integrate the tactile displays into information devices, it is mandatory that the displays are thin and offer sufficient flexibility. Further, the mechanical parts should be minimized to protect the displays from external damages.

The principle of electrovibration tactile display was accidently discovered by Mallinckrodt et al. [18]. The electrovibration tactile displays directly modulate frictional force via electrostatic force and provide mechanical vibration stimulus to the user. Thus, they can provide a more natural tactile stimulus to the users. The electrovibration tactile displays have been studied for several decades [19,20,21]. The tactile display requires only an electrode and an insulator layer, and it can be thin and even transparent. Thus, it has good compatibility with conventional information devices. Using a polyethylene terephthalate (PET) substrate and an indium tin oxide (ITO) electrode, the display can be made thin and flexible. The electrovibration tactile displays do not require mechanical parts, and have sufficient durability against external damages. However, PET is not perfectly flexible [22,23]. ITO is a metal oxide material, and has mechanical brittleness [24,25]. A high vacuum level and high temperature are required for the sputtering process of ITO [26,27]. Hence, the electrovibration tactile display that consists of PET substrate and ITO electrode is not perfectly flexible. The electrodes are likely to crack under excessive bending when the electrode is not spatially activated, and the electrovibration stimulus is not provided on the entire surface of the electrovibration tactile display.

**Figure 1:**
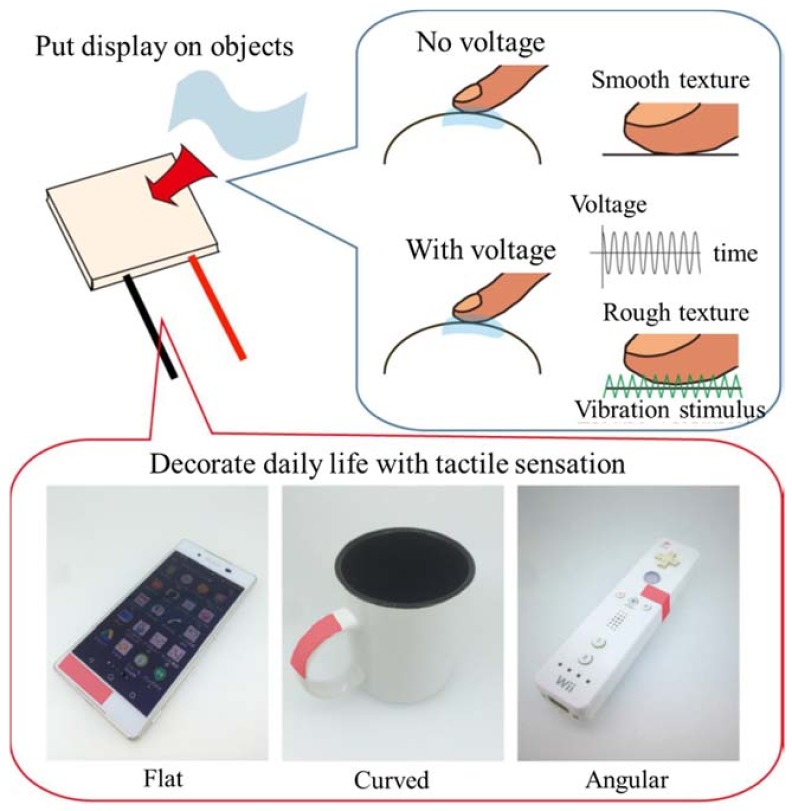
Concept of the proposed tactile display. The tactile display is based on electrovibration stimulus; it provides tactile sensation with applied voltage. It is sufficiently flexible so that it can be attached onto various surfaces.

In this study, a sheet-type electrovibration tactile display, which is sufficiently thin and flexible to be attached onto the surfaces of various devices, is demonstrated. The concept of this display is shown in Figure 1. Polydimethylsiloxane (PDMS), which is transparent and a few hundred times more flexible than PET, is used to form the substrate and the insulator layer [28]. The dielectric constants of PDMS and glass are almost of the same order, and it is considered that the PDMS based insulator can present electrovibration stimulus to the users [29,30]. A poly(3,4-ethylenedioxythiophene) polystyrene sulfonate (PEDOT/PSS) is used to form the electrode. PEDOT/PSS is a transparent conductive polymer and its elastic modulus is high [31]. However, it is known that this material is highly robust under bending and stretching [32,33]. Thus, the proposed tactile display is fully flexible and is durable under bending. In addition, only spin-coating and baking under atmospheric pressure are required to process this material. Thus, the cost would be lower and damages during fabrication can be prevented. The thickness of the tactile display can be controlled by the spinning speed during the coating process, and hence the display can be made sufficiently thin by spinning at a high speed. Bau et al. also proposed a similar method to add tactile sensation to familiar devices with electrovibration tactile stimulus [34]. The layer for tactile stimulus was formed by painting conductive and insulation materials, which resulted in non-uniform thickness. Since the intensity of the electrovibration depends on the thickness of the insulation layer, the painting process resulted in spatial non-uniformity of the tactile stimuli. The device proposed in this study possesses uniform thickness of the layer, and hence it provides uniform tactile stimuli. Additionally, the proposed tactile display can be attached and removed easily. First, the display was fabricated and its robustness under bending was proved. Next, the tactile stimuli presented by the display were characterized. Previous studies on the evaluation of electrovibration tactile displays focused on threshold voltage evaluation [19,20,21,34,35]. The tactile sensations presented, which are important for tactile rendering, have not been sufficiently investigated in these studies. Thus, a sensory evaluation was conducted with subjects to characterize the proposed tactile display. Finally, the effect of a combination of two tactile stimuli was demonstrated to verify the effectiveness of the proposed display.

## 2. Principle

**Figure 2:**
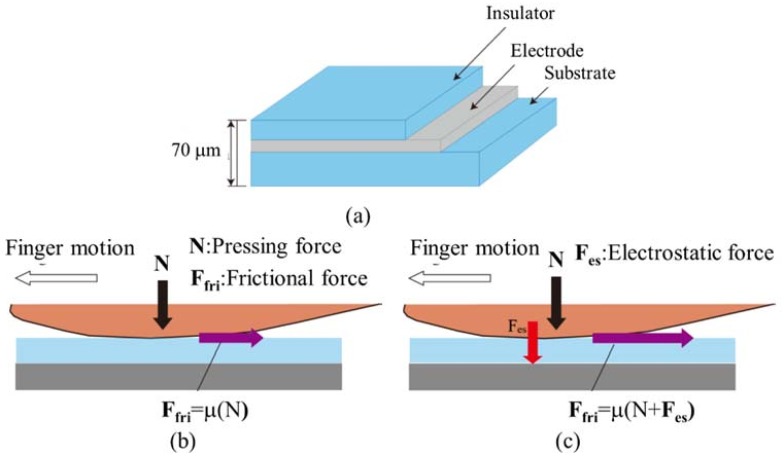
Conceptual design and principle of the proposed tactile display. (**a**) Schematic view of the tactile display, which consists of the polydimethylsiloxane (PDMS) layer and the polystyrene sulfonate (PEDOT/PSS) layer; (**b**) Frictional force without voltage; (**c**) Frictional force with voltage. Electrostatic force is applied to the contacting skin. As a result, the fictional force increases.

Figure 2 shows the principle of the proposed tactile display and its use for electrovibration stimulus. A transparent and flexible PEDOT/PSS electrode is sandwiched between a PDMS substrate and a PDMS insulator layer, as shown in Figure 2a. The thickness of the substrate and the insulator layer are 60 μm and 10 μm, respectively. The spin-coated PEDOT electrode is less than 1 μm in thickness [36,37]. Thus, the total thickness of the tactile display is approximately 70 μm. The PEDOT electrode is connected to a high voltage power supply. Users scan the surface of the display with their finger. When no voltage is applied, no external force is exerted on the contacting finger and the users perceive a flat surface, as shown in Figure 2b. When voltage is applied to the electrode, it is charged positively and the finger is charged negatively. Thus, an electrostatic force is generated between them. This attracts the finger toward the electrode and displays tactile sensation or surface textures to the users, as shown in Figure 2c. The electrostatic force can be expressed as follows [38].

(1) $$F=\frac{A\varepsilon \varepsilon_{0}}{2}{\left(\frac{V^{\prime }\left(t\right)}{d}\right)}^{2}$$

where *F* is the electrostatic force that acts as the attractive force, *ε*_0_ is the vacuum permeability, *ε* is the relative permeability of the stratum corneum, *A* is the contacting area of the fingerpad, *V’*(*t*) is the applied voltage across the stratum corneum, and *d* is the thickness of the stratum corneum. The waveform of the voltage across the stratum corneum is different from that of the applied voltage, since the applied voltage is filtered by the equivalent circuit formed by the body and the electrovibration tactile display. The resulting frictional force can be expressed as follows.

(2) $$F^{\prime }=\mu \left(F+N\right)=\mu \left(\frac{A\varepsilon \varepsilon_{0}}{2}{\left(\frac{V^{\prime }\left(t\right)}{d}\right)}^{2}+N\right)$$

where *F*’ is the resulting frictional force, *N* is the force that the users originally applied on the surface by pressing, and *μ* is the friction coefficient of the surface. Equation (2) shows that the frictional force changes with the applied voltage. In electrovibration, pulse or sinusoidal voltage is typically applied to the electrode. The periodic change in voltage results in a similar periodic change in the frictional force. Consequently, the skin is periodically deformed by the frictional force and the users can perceive the vibration stimulus while scanning the surface. Thus, the surface textures depend on the applied voltage. Moreover, a high peak voltage is required to induce fictional force that is sufficient to stimulate mechanoreceptors.

## 3. Materials and Methods

### 3.1. Materials

A PDMS casting solution (Sylpot 184, Dow Corning Toray, Tokyo, Japan) was used to form a substrate and an insulator layer. PDMS is a thermosetting polymer and has a Young’s modulus of 2.5 MPa and dielectric constant of almost 3 [28,29]. A fluorine resin (CYTOP CTX, Asahi glass, Tokyo, Japan) was used to reduce the adhesion force between glass substrate and PDMS. PEDOT/PSS (768842-25G, Sigma Aldrich Co. LLC., St. Louis, MI, USA) was used as the material for the electrode. It has a Young’s modulus of 3 GPa [30].

### 3.2. Fabrication Process

**Figure 3:**
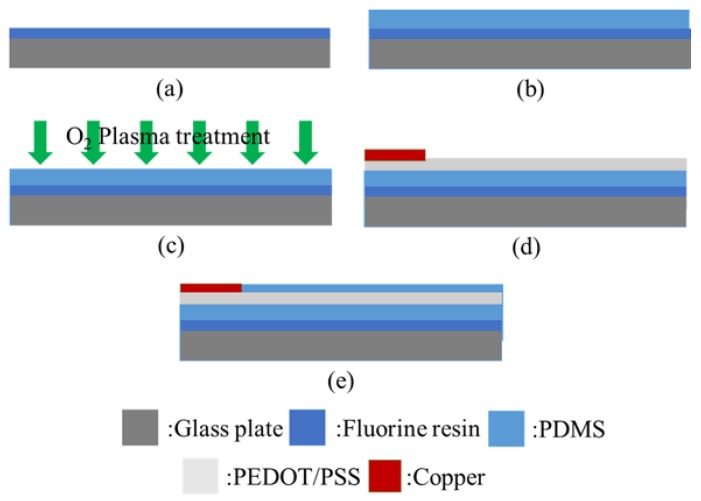
Fabrication process of the proposed tactile display. (**a**) Fluorine resin layer formation; (**b**) PDMS substrate formation; (**c**) O_2_ plasma treatment; (**d**) PEDOT/PSS electrode formation and copper electrode attachment; (**e**) Insulation layer formation.

**Figure 4:**
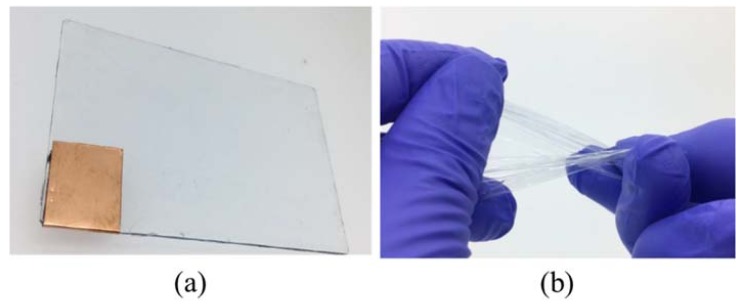
Images of the fabricated tactile display. (**a**) Fully flexible and transparent tactile display; (**b**) Twisting of the tactile display. The tactile display can be easily twisted by hand because of its flexibility.

Figure 3 shows the fabrication process of the tactile display. A glass substrate was spin-coated with a fluorine resin at 1000 rpm for 40 s. A PDMS casting solution was spin-coated on the glass substrate at 1000 rpm for 20 s and baked at 100 °C for 10 min to form a PDMS substrate. The PDMS substrate was exposed to Oxygen plasma to improve wettability, as shown in Figure 3c. Then, a PEDOT/PSS solution was spin-coated at 500 rpm for 20 s and was baked at 100 °C for 10 min for curing. To connect the tactile display easily to a high voltage power supply, a copper tape was attached onto the PEDOT/PSS electrode, as shown in Figure 3d. This was followed by spin-coating a PDMS casting solution at 5000 rpm for 40 s and baking at 100 °C for 10 min to form the insulation layer on the electrode, as depicted in Figure 3e. Finally, the remaining PDMS layer on the copper electrode was removed with a cutter knife. Figure 4a shows the fabricated tactile display. Since the PDMS layer and PEDOT/PSS electrode were highly transparent, the tactile display was also transparent. The tactile display was used after it was released from the glass plate. As shown in Figure 4b, the released tactile display could be easily folded or bent.

### 3.3. Experimental Procedures

#### 3.3.1. Bending Experiments

There is a strong requirement that it should be possible to attach the tactile display onto a curved surface, while the excessive deformation may result in fracture of the PEDOT/PSS electrode. The cracks in the electrode would lead to inadequate charge on the entire surface area of the electrode. The durability of the tactile display under bending was evaluated. The tactile display was cut with a cutter knife to a size of 15 mm × 60 mm. It was then attached onto curved acrylic surfaces with curvature radii in the range of 5–20 mm. The acrylic cylinders were manufactured with a computer numerical control (CNC) miller (MM-100, Modia Systems, Tokyo, Japan). The change in resistance, which depends on the cracks in the PEDOT/PSS electrode, was measured. The procedure was repeated five times. In this experiment, copper tape electrodes were attached on both ends to measure the change in resistance.

#### 3.3.2. Effect of Voltage Waveform on Perception

**Figure 5:**
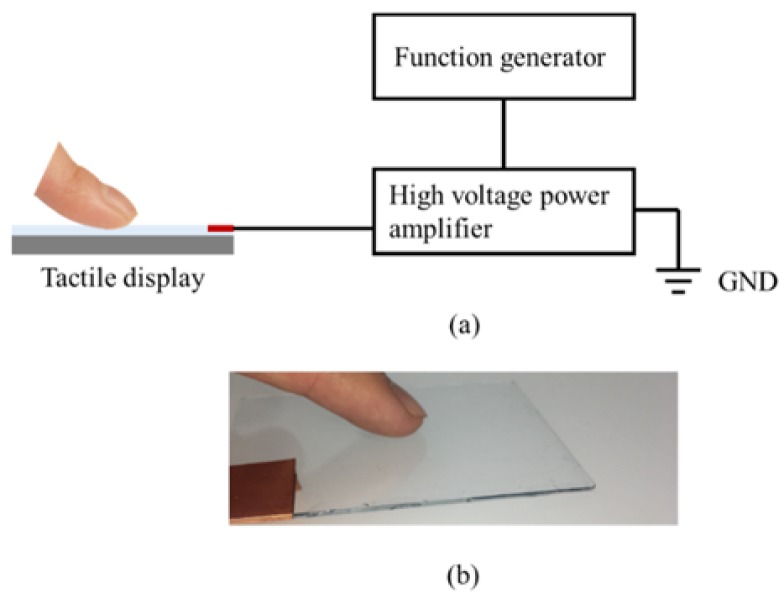
(**a**) Schematic illustration of the experimental setup; (**b**) Image of the experimental setup. The experimental setup consisted of the tactile display, function generator, and high power amplifier. The ground (GND) of the high voltage power amplifier was connected to the desk.

Equation (2) shows that the frictional force depends on the voltage waveform. The relationship between the voltage waveform and perception was evaluated through experiments with five subjects (five males in their 20s). This experiment was approved by the Research Ethics Committee of Faculty of Science and Technology, Keio University. The experimental setup consisted of a tactile display, a high voltage power amplifier (HSA4052, NF Corporation, Tokyo, Japan), and a function generator (33210A, Agilent Technology, Santa Clara, CA, USA), as shown in Figure 5a. The voltage waveform applied to the tactile display was determined by the voltage signal from the function generator. The ground (GND) was connected to the desk where the experimental setup was fixed. The tactile display was attached onto a glass plate, and the subjects scanned the display with their index finger without any applied voltage for 10 s. Then, a voltage with a different waveform was applied for 10 s while the subjects scanned the tactile display. The scanning speed was not controlled, considering the conditions in practical applications. The subjects were asked to answer whether they were able to distinguish between the two surfaces, i.e., with and without the applied voltage. They were also asked to describe how they perceived the stimulus. The applied voltage waveforms were sinusoidal, triangle, and square waves with a duty cycle of 50%. The frequencies were 5, 10, 50, 100, 200, 300, and 400 Hz. The peak voltage was fixed at 250 V. A high value was selected for the peak voltage, since a low peak voltage caused low intensity of electrovibration stimulus. The experimental conditions were selected randomly. Figure 5b shows the image of the experimental setup. It was ensured that each subject cleaned their index finger before the commencement of the experiment. The number of the correct answers were counted and the figure was divided by the number of trials to calculate the rate of perception.

#### 3.3.3. Waveform Discrimination

Investigation was conducted with ten subjects (ten males, age 20s) to understand how the surface textures generated by the different voltage waveforms could be discriminated. The experimental setup was the same as the one described in Section 3.3.2. First, a voltage waveform was applied to the tactile display. Then, the subjects scanned the tactile display with their index finger for 10 s. Subsequently, several voltage waveforms, including the voltage waveform selected initially, were provided. The subjects were asked to scan the tactile display surface for 10 s. They were asked to select the same stimulus as the first one. The tested voltage waveforms were sinusoidal, triangle, and square waves, and all the waveforms had a duty cycle of 50%. The frequency was 50 Hz, and the peak voltage was fixed at 250 V. The experimental condition was selected randomly. In each condition, the experiment was conducted six times, and each subject was made to clean his index finger before the experiment began. The experimental conditions are presented in Table 1. The total number of correct answers were counted, and the figure was divided by the number of trials to calculate the rate of discrimination.

#### 3.3.4. Effect of Duty Cycle

The effect of a duty cycle was examined, in particular, with respect to surface roughness. Five subjects participated in the experiments (five males, age 20s). The experimental setup was the same as the one described in Section 3.3.2. The subjects scanned the tactile display with their index fingers. They were asked to provide a response on the perceived roughness, which was labeled from 0 to 6. A square voltage waveform was applied. The frequencies were 10, 50, 100, and 200 Hz. The duty cycles were 20%, 40%, 60%, and 80%. The peak voltage was fixed at 250 V. The voltage condition was selected randomly and each subject cleaned his index finger before the experiment. The average of the responses under each condition was calculated to evaluate the results.

#### 3.3.5. Multiple Tactile Stimulus

**Figure 6:**
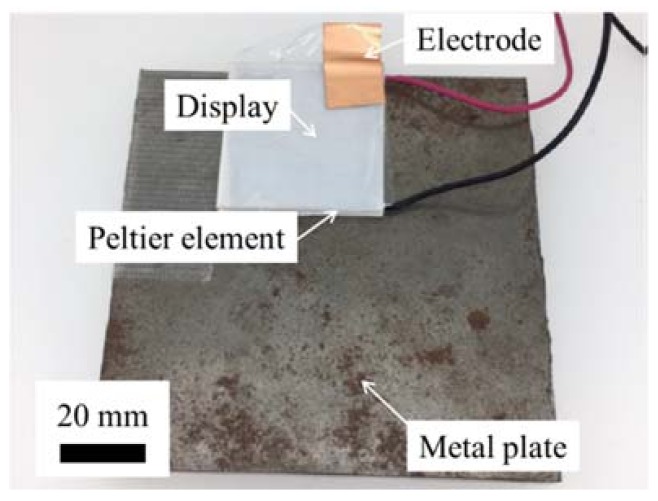
Experimental setup of the combined tactile stimuli. The tactile display was attached onto a Peltier element. The tactile display was connected to the high voltage power supply which was controlled by the function generator. The Peltier element was connected to the DC power supply.

Owing to the thin and flexible structure of the proposed electrovibration tactile display, it can be integrated with different types of tactile displays. In this work, we attempted to combine it with a thermal display, where the thinness is beneficial for heat transfer. To the best of our knowledge, multiple tactile stimuli of electrovibration stimulus and thermal stimulus have never been investigated. Thus, an evaluation was performed to check whether the combined tactile stimulus can be discriminated by subjects. The integrated tactile display is shown in Figure 6. The electrovibration tactile display was attached onto a Peltier element (TEC1-12706, Hebei I.T., Shanghai, China) and connected to a function generator (33210A, Agilent Technology, Santa Clara, CA, USA) and a high voltage power amplifier (HSA4052, NF Corporation, Yokohama, Japan). The Peltier element was connected to a DC power supply (AD-8723D, A&D Corporation, Tokyo, Japan) and fixed on a metal plate for cooling. The effect of the multiple tactile stimuli was evaluated with ten subjects (10 males, age 20s). Initially, the subjects scanned the tactile display under a certain stimulus condition and then several conditions including the original condition were presented to the users for 10 s. The subjects were asked to select the same one as the original condition. The temperature of the Peltier element was set to either 20 °C or 30 °C, and the temperature was monitored with a noncontact temperature sensor (MT-006, Mothertool Corporation, Ueda, Japan). Square voltage waveform, with a duty cycle of 50%, was applied. The frequencies of the voltage were 10 Hz and 100 Hz. The peak voltage was fixed at 250 V. The electrovibration and temperature condition were selected randomly. Each condition was tested eight times; the experimental conditions are presented in Table 2. The number of correct answers were counted, and the figure was divided by the number of trials to calculate the rate of discrimination.

## 4. Experimental Results

### 4.1. Bending Experiments

**Figure 7:**
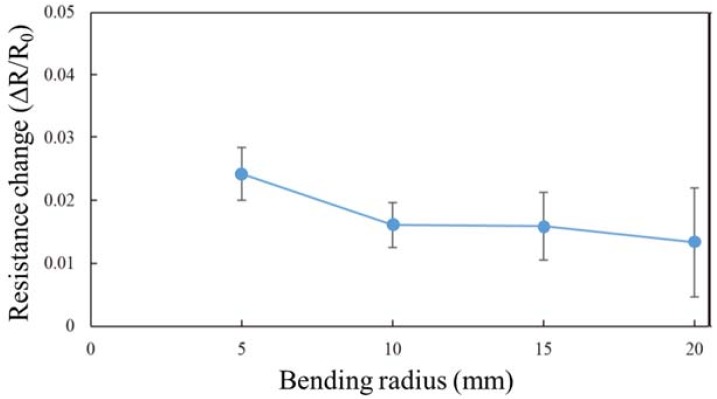
Relationship between bending radius and resistance change. The error bars represent standard deviations. The change in resistance decreases with the increase in bending radius.

Figure 7 shows the experimental results on the effect of bending radius. The resistance of the PEDOT/PSS electrode was measured at different bending radii. The results indicate that the PEDOT/PSS electrode was successfully formed with the proposed fabrication process. The change in resistance decreased with increase in the radius of curvature. Cho et al. [30] reported that the resistance of the PEDOT/PSS electrode increased with a radius of curvature of 5 mm or less, which is similar to the trend obtained in this study. The change in resistance was less than 3%. Thus, it can be inferred that no severe crack occurred in the electrode. Small cracks in the electrode or stretching of the tactile display resulted in a small change in the resistance.

### 4.2. Effect of Voltage Waveform

**Figure 8:**
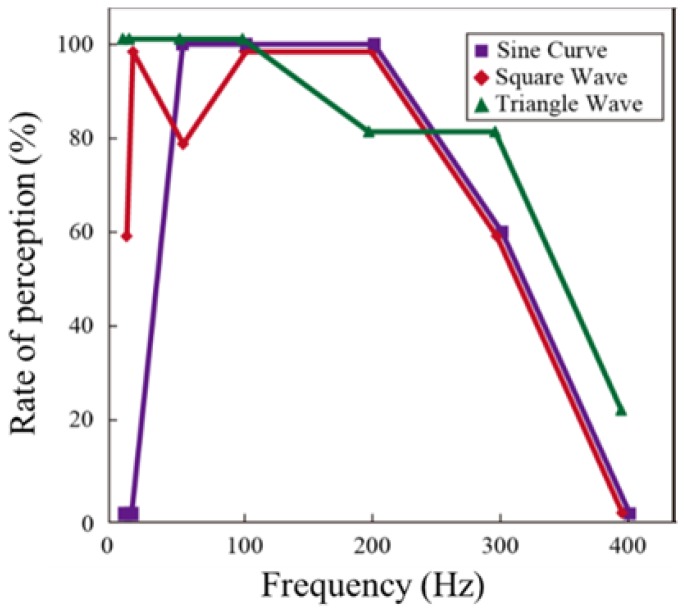
Relationship between frequency and rate of perception. The trend is similar for each voltage waveform. The rate of perception is high at low frequencies and low at high frequencies.

Figure 8 shows the relationship between the frequency of the applied voltage and successful rate of perception. The rate of perception was relatively high at low frequency for almost all the voltage waveforms. However, the subjects were not able to perceive the sinusoidal wave at 5 Hz. Varder et al. reported that the simulated input voltage applied to the skin decreased significantly under low frequency sinusoidal waveform conditions and that the resulting electrostatic force also decreased significantly [38]. It is believed that the decrease in electrostatic force under low frequency condition resulted in a low rate of perception and this rate also decreased with increase in the frequency. Bau et al. reported that the subjects perceived smooth stimulus under sinusoidal waveform at 400 Hz [35]. At high frequency, rubbery tactile sensation is developed by the frictional force of PDMS, and this has a stronger influence on perception than electrovibration stimulus. The response for the tactile sensation of the sinusoidal waveform could either be “slippery” or “smooth.” The amplitude of the calculated electrostatic force was low under sinusoidal wave condition [35]. Thus, the subjects answered with these words, which indicated a smooth surface. On the other hand, the response for the tactile sensation of pulse voltage and triangle waveform was either “bumpy” or “rough.” In the case of pulse voltage, the amplitude of the calculated electrostatic force was higher than that of the sinusoidal voltage and was edgy in nature [38]. This trend resulted in the above answers for the tactile sensation. The same trend was observed for the triangle waveform also.

### 4.3. Waveform Discrimination

**Figure 9:**
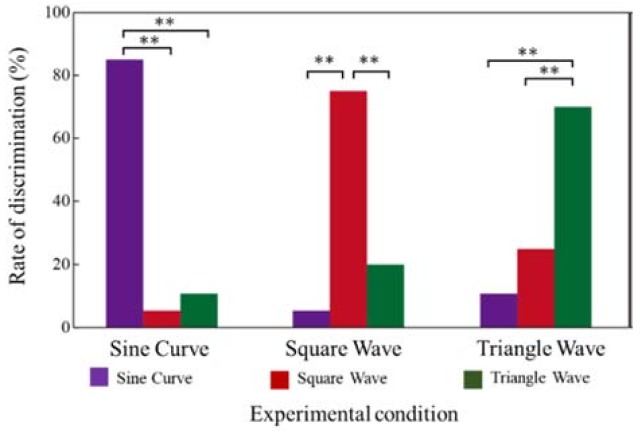
Relationship between the experimental condition and rate of discrimination. “**” means 1% level of significance. Each waveform was well discriminated from the other waveforms. The results indicate that each voltage provided a unique tactile sensation.

Figure 9 shows the experimental results on waveform discrimination. The subjects were able to discriminate one stimulus from the other stimuli with a rate of over 70%. To evaluate the results statistically, chi-square test was also conducted for each set of conditions. The results showed that the subjects were able to accurately discern each voltage waveform from the other waveforms with a 1% level of significance. Each voltage waveform caused unique frictional force modulation that was apparently different for the other waveforms. The waveform control is an effective method to control the tactile sensation presented on the tactile display. However, some subjects answered that the pulse voltage and triangle waveform presented a similar tactile sensation. It was concluded that the electrostatic force is edgy and has a similar trend for both pulse voltage and triangle waveform. As a result, the subjects occasionally misidentified similar waveforms.

### 4.4. Effect of Duty Cycle

**Figure 10:**
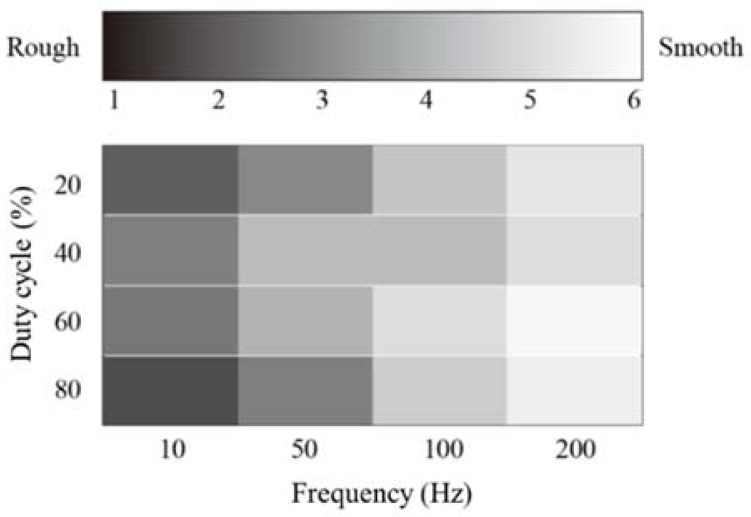
Relationship between voltage condition and perceived roughness. The roughness level is shown in gray scale. The subjects perceived rough surface at low frequencies and smooth surface at high frequencies. The level of the roughness can be controlled by changing the duty cycle.

Figure 10 shows the relationship between the voltage waveform and perceived roughness. The level of perceived roughness increased with frequency. The frictional force changed rapidly at high frequency. Hence, the contacting skin was also subjected to rapid vibrations. The vibration condition of the skin was similar to that when the subjects stroked fine and smooth surfaces. Thus, the subjects perceived smooth tactile sensation under high frequency conditions. However, the frictional force changed slowly under low frequency conditions; this gave the perception of a rough and bumpy surface. The electrovibration stimuli at duty cycles of 20% and 80% were perceived as rougher surfaces. From the results, it can be inferred that the duty cycle also influences the perceived roughness. When the ratio of high frictional force to low frictional force is extremely high or low, the subjects perceived a rough surface. The electrovibration stimulus is caused by the change in the frictional force; when this type of stimulus has a higher and lower duty ratio, it causes a rapid change in the frictional force. Therefore, the skin surface vibrates rapidly and the perceived intensity of the electrovibration stimulus increases; this is perceived as a rough surface.

### 4.5. Multiple Tactile Stimulus

**Figure 11:**
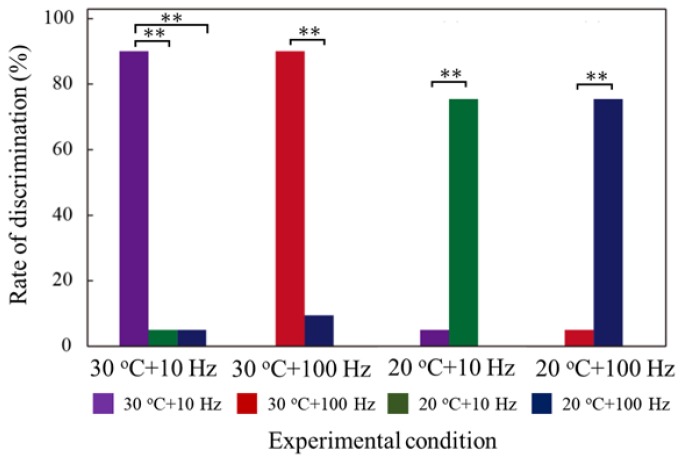
Relationship between multiple tactile stimuli condition and rate of receiving correct answers. “**” means 1% level of significance. Each multiple stimulus can be discriminated from the other stimuli. Multiple tactile stimuli condition is effective in improving the range of the tactile sensation presented.

The relationship between the experimental condition and the rate of receiving correct answers is shown in Figure 11. Chi-square test was also conducted to statistically evaluate the results. From the analysis, it can be seen that the subjects could discriminate each stimulus condition with 1% level of significance. The subjects discriminated one stimulus condition from the other conditions with better accuracy when the intensity of each stimulus condition was significantly different. However, they occasionally misidentified the temperature condition, resulting in a low rate of correct answers. It appears that the electrovibration stimulus might have more influence on the tactile perception than the thermal stimulus. From the experimental results, it can be inferred that the electrovibration stimulus provided by the proposed tactile display has the potential to be combined with thermal stimulus under the simplified experimental condition, leading to a more realistic tactile sensation.

## 5. Conclusions

The above experimental results show that the proposed tactile display can be attached onto curved surfaces and that it can provide tactile stimulus. The proposed structure has good durability under bending. Electrical disconnection does not occur under bending condition when the radius of curvature is greater than 5 mm. Thus, the tactile display can provide tactile sensation on the entire surface under bending condition. Further, the tactile display might have the potential to provide tactile sensation under bending condition with a radius of curvature greater than a few mm or less. The electrode is not electrically disconnected until the resistance of the electrode becomes infinite. For practical applications, the relationship between mechanical bending and the tactile sensation provided needs to be clarified. The results of the study show that the tactile stimulus provided can be successfully perceived by the subjects. The applied voltage waveform affected the tactile stimulus significantly. The frequency of the voltage waveform is important to provide a clear tactile sensation. The subjects were able to perceive the tactile stimulus under high frequency condition. This indicates that the electrovibration stimulus is detected by mechanoreceptors, such as Meissner corpuscles and Pacinian corpuscles. To provide a clear tactile sensation through the tactile display, the characteristics of these mechanoreceptors should be considered [39]. Further, the shape of the voltage waveform is related to the intensity and perception of the electrovibration stimulus. It is assumed that the rapid change in the applied voltage waveform caused an increase in the electrovibration stimulus. Although the relationship between the shape of the electrovibration stimulus and the perception has not been revealed in terms of biomechanics, the shape of the voltage waveform is also an important factor for the electrovibration stimulus. Multiple tactile stimuli of the electrovibration and thermal types were evaluated, since the thinness of the tactile display is effective in improving heat transfer. The multiple tactile stimuli were successfully distinguished by the test subjects. However, the electrovibration and thermal stimulus were largely differed. Thus, to characterize the multiple stimulus, a detailed evaluation of the multiple stimuli is required to characterize the stimuli.

Further study is required to implement the proposed tactile display. One problem area is the power supply. The large high-voltage power supply used in this study is not suitable for attaching to other devices. Hence, the power supply unit has to be minimized with a small booster circuit, or the structure, material, and voltage waveform should be optimized to provide electrovibration stimulus under low voltage amplitude condition. In addition, more sensory evaluations are also required to characterize the proposed electrovibration stimulus. The detailed characterization will help determine the fields where the proposed tactile display can be applied.

## Figures and Tables

**Table 1:** Experimental conditions for the discrimination experiment.

Experimental Condition			
	I	II	III
Waveform	Sinusoidal	Square	Triangle
Voltage	250 V	250 V	250 V
Frequency	50 Hz	50 Hz	50 Hz

**Table 2:** Experimental conditions for the multiple tactile stimuli evaluation.

Experimental Condition				
	I	II	III	IV
Surface temperature	30 °C	30 °C	20 °C	20 °C
Peak voltage	250 V	250 V	250 V	250 V
Frequency	10 Hz	100 Hz	10 Hz	100 Hz
